# SFTA2—A Novel Secretory Peptide Highly Expressed in the Lung—Is Modulated by Lipopolysaccharide but Not Hyperoxia

**DOI:** 10.1371/journal.pone.0040011

**Published:** 2012-06-29

**Authors:** Rashmi A. Mittal, Markus Hammel, Johannes Schwarz, Katharina M. Heschl, Nancy Bretschneider, Andreas W. Flemmer, Susanne Herber-Jonat, Melanie Königshoff, Oliver Eickelberg, Andreas Holzinger

**Affiliations:** 1 Neonatology, Dr. von Haunersches Kinderspital, Ludwig-Maximilians-Universität München, Munich, Germany; 2 Comprehensive Pneumology Center, Institute of Experimental Pneumology, University Hospital, Ludwig-Maximilians-Universität München and Helmholtz Zentrum München, Munich, Germany; 3 Genomatix Software GmbH, Munich, Germany; University of Crete, Greece

## Abstract

Tissue-specific transcripts are likely to be of importance for the corresponding organ. While attempting to define the specific transcriptome of the human lung, we identified the transcript of a yet uncharacterized protein, SFTA2. *In silico* analyses, biochemical methods, fluorescence imaging and animal challenge experiments were employed to characterize SFTA2. Human *SFTA2* is located on Chr. 6p21.33, a disease-susceptibility locus for diffuse panbronchiolitis. RT-PCR verified the abundance of *SFTA2*-specific transcripts in human and mouse lung. SFTA2 is synthesized as a hydrophilic precursor releasing a 59 amino acid mature peptide after cleavage of an N-terminal secretory signal. SFTA2 has no recognizable homology to other proteins while orthologues are present in all mammals. SFTA2 is a glycosylated protein and specifically expressed in nonciliated bronchiolar epithelium and type II pneumocytes. In accordance with other hydrophilic surfactant proteins, SFTA2 did not colocalize with lamellar bodies but colocalized with golgin97 and clathrin-labelled vesicles, suggesting a classical secretory pathway for its expression and secretion. In the mouse lung, *Sfta2* was significantly downregulated after induction of an inflammatory reaction by intratracheal lipopolysaccharides paralleling surfactant proteins B and C but not D. Hyperoxia, however, did not alter SFTA2 mRNA levels. We have characterized SFTA2 and present it as a novel unique secretory peptide highly expressed in the lung.

## Introduction

A number of lung diseases are likely to be caused by genetic defects but their pathogenesis is not yet understood. In order to identify human genes predominantly expressed in the lung, we screened public databases containing tissue expression profiles. We identified *SFTA2* as a gene specifically expressed in the lung. A partial mRNA corresponding to the human transcript had been submitted to NCBI GenBank by M.G. Walker and P. Spiro in 2002 (Accession AY102070) as “co-expressed with pulmonary surfactants”. There is, however, no literature associated with this finding. The official gene symbol is *SFTA2* (‘Surfactant associated 2′; HUGO Nomenclature Committee; [1]). Although there is no published report focusing on *SFTA2*, Clark et al. [2] predicted secretion signals for over 1000 proteins from public databases, SFTA2 being among them. Zhang and Henzel [3] tested the ability of a computer algorithm to predict cleavage sites of secretory signals in 270 secreted proteins. For SFTA2 it was found, that a 19 amino acid signal at the amino terminus was cleaved off from a precursor. However, as yet there are no published data on expression or function of *SFTA2*. Here we present the identification of *SFTA2* as a gene preferentially expressed in the lung as well as detailed expression, biochemical and regulation data.

## Methods

### *In Silico* Identification and Characterization of SFTA2

Human tissue expression patterns of GEO Profiles (http://www.ncbi.nlm.nih.gov/geoprofiles) and GeneNote [4] were screened to identify genes preferentially expressed in the lung [5]. Homology searches and multiple alignments used BLAST from the National Center for Biotechnology Information. Gylcosylation and signal prediction used the NetNGlyc 1.0 and SignalP 3.0 servers respectively (Technical University of Denmark). The PSIPRED Protein Structure Prediction Server (University College London) and the DiANNA software (identification of potential disulfide bonds [6]) were also employed.

### Expression Analysis of Normal Tissues by RT-PCR

TissueScan® qPCR cDNA array analysis (Origene, Rockville, MD, USA) was performed on 48 human tissues using *SFTA2*-specific primers according to the manufacturer. The data were normalized for glyceraldehyde-3-phosphate dehydrogenase (GAPDH) or β-actin. In the mouse, RNA was extracted from 10 normal tissues (lung, liver, heart, kidney, spleen, uterus, cerebrum, cerebellum, adrenal gland, small intestine), and qPCR-analysis was performed with normalization for actin on a Mastercycler® ep realplex (Eppendorf, Hamburg, Germany). Primers used were- forward: CACACGCAGGGCCAAAGGTG and reverse: GAGGAGGCAGATCTTTTGGAG.

### *In Silico* Promoter Analysis

Promoter sequences for *SFTA2* homologues from 8 species (macaca mulatta, pan troglodytes, homo sapiens, mus musculus, canis lupus familiaris, bos taurus, sus scrofa and equus caballus) were extracted from the ElDorado database and aligned using DiAlign [7]. Searches for conserved transcription factor binding sites and combinations of these (models) were carried out using FrameWorker (Matrix library 8.3). Common patterns were required to be present in at least 7 of 8 promoter sequences, with distances between 10 and 200 bp between neighbouring binding sites, and with no more than 10 bp distance variation. ModelInspector was used to scan the *SFTA2* promoters for known functional (published) modules and all human promoter sequences for newly defined models from *SFTA2*. DiAlign, FrameWorker and ModelInspector are part of the Genomatix Software Suite (www.genomatix.de).

### Generation of Expression Constructs

A human *SFTA2* cDNA clone (IRATp970H02140D) from Source BioScience ImaGenes (Berlin, Germany) served to generate expression plasmids by PCR. The vector pCDNA3.1 (Invitrogen) was used to generate an expression plasmid containing a C-terminal HA epitope tag (pCDNA-SFTA2-HA). The vector pMal-c2 (New England Biolabs) was used to generate a construct for bacterial expression of maltose-binding protein (MBP) fused to SFTA2 (MBP-SFTA2) excluding the signal sequence. MBP-SFTA2 was induced and prurified from DH5α E. coli according to the manufacturer.

### Transfection of Human and Mouse Cell Lines and Immunofluorescence Microscopy

A549 cells (human lung adenocarcinoma) and MLE12 cells (mouse lung epithelium) were grown to 80% confluence and transfected using the Amaxa Nucleofector device (Lonza Cologne GmbH, Cologne, Germany). For immunofluorescence microscopy, cells were plated on coverslips, fixed with 3% paraformaldehyde for 15 min., permeabilized with 0.15% Triton X-100 for 15 min. and exposed to primary antibodies in a concentration of 1∶100. The HA–tag was detected using the high affinity rat antibody clone 3F10 in a dilution of 1∶100 (Roche Diagnostics Mannheim, Germany). A549 and MLE12 cells have been used previously to address the issue of subcellular targeting to lamellar bodies [8], [9]. Lamellar bodies were detected by labeling with anti-LAMP-1 antibodies in A549 cells (clone H4A3) from Development Studies Hybridoma Bank (Iowa, USA.). In MLE12 cells, we labelled lamellar bodies by transfection of ABCA3 (ATP binding cassette subfamily A member 3) cDNA (encoding a lamellar body lipid transporter) carrying a C-terminal HA-epitope tag. Golgi bodies were detected using golgin-97 antibody in a dilution of 1∶200 (Invitrogen, Life Technologies, Grand Island, U.S.A.). Clathrin-labelled vesicles were detected by labelling with anti-Clathrin antibody clone X22 in a dilution of 1∶200 (Abcam, Cambridge, U.K.). Secondary antibodies were compatible goat anti-species IgG labeled with Alexa fluorophores (Invitrogen, Carlsbad, USA). 4′,6-diamidino-2-phenylindole (DAPI) was used for counterstaining nuclei. Immunofluorescent cell imaging was performed with a Zeiss Axiovision 135 inverse fluorescence microscope.

### Generation of Specific Antibodies against Human SFTA2

Three overlapping peptides corresponding to the human sequence (LKLKESFLTNSSY**c**, EKLCLLLHLPSGTS**c**, SGTSVTLHHARSQHHV**c**) were coupled to KLH. Two rabbits were immunized; each with a combination of all three conjugates. Serum was harvested and tested in ELISA for a specific response against uncoupled peptides. One rabbit mounted a significant response against all peptides. This serum was purified against all three peptides immobilized to a matrix (Primm, Milano, Italy).

### Western Blot and Glycosylation Testing

Cell lysates were prepared with RIPA (radioimmunoprecipitation) lysis buffer. Immunoblot analysis followed standard procedures. HA-antibody as above was used in a dilution of 1∶1000, while peptide antibodies were used 1∶250 to 1∶500. Detection was achieved by appropriate goat anti IgG antibodies conjugated to horseradish peroxidase (1∶7500) and SuperSignal® West Femto detection system (Thermo Scientific, Rockford, IL, USA). In order to test for N-linked glylcosylation we used Endo H on lysates of cells transfected with pcDNA-SFTA2-HA according to the manufacturer (New England Biolabs).

### Immunostaining of Human Lung Tissue

The use of anonymous human lung tissue was approved by the ethics committee of the Ludwig-Maximilian University, Munich, Germany (Reference number: EK 15032011). We were given permission for use of anonymized lung tissue (left over after clinical investigations) without consent. Lung tissue had been freshly collected during bronchoscopy, snap-frozen in liquid nitrogen, and stored at −80°C. For immunostaining, frozen sections (4 µm) were prepared (Frozen Section Medium Neg-50; Richard-Allan Scientific, MI, USA), fixed with cold acetone for 2 minutes, dried and then stored at −80°C. After abolishment of endogenous peroxidase activity (3% H_2_O_2_, 15 minutes) sections were treated with primary antibodies specific for thyroid transcription factor-1, (TTF-1 1:50; mouse monoclonal 8G7G3/1; Santa Cruz Biotechnology, CA, USA) and SFTA2 (1∶50; rabbit; purified peptide antibody) for 60 minutes at room temperature. Detection was achieved with compatible fluorescein-conjugated secondary Alexa Fluor antibodies (Invitrogen, CA, USA). Nuclei were counterstained with DAPI (05 µg/ml; Sigma-Aldrich, Germany) and covered with fluorescent mounting medium (DAKO; Glostrup, Denmark). The preparations were analyzed with a Zeiss Axio Imager fluorescence microscope (Zeiss; Munich, Germany) and images were captured with a monochromatic Zeiss Axiocam and Axiovison software (Version 4.8). Differential interference contrast microscopy enabled visualization of lung morphology.

### Animal Experiments

All animal experiments were performed according to the Federal Act on the Protection of Animals (Germany) and approved by the committee of the District Government of Upper Bavaria (Reference number: 55.2-1-54-2531-24-08). For the lipopolysaccharide (LPS)-challenge study, fourteen 9–11 week-old female C57BL/6 mice were anesthetized by intraperitoneal injection of Medetomidin, Midazolam and Fentanyl and randomized to receive an intratracheal instillation of either 1 mg/kg body weight LPS (LPS O55:B5, Sigma, Munich, Germany) in normal saline (total volume 80 µl) or 80 µl normal saline. Immediately after instillation, the lungs were inflated with a Flexivent small animal ventilator (DMI, Cerritos, CA, USA) to allow for even distribution. The animals were then kept as usual for 18 h after which they received an intraperitoneal injection of pentobarbital and were killed by exsanguination. Following tracheotomy, the lungs were lavaged twice with 0.6 ml of normal saline and the recovered fluid was pooled. Then the lungs were removed, snap-frozen in liquid nitrogen and stored at −80°C.

For the oxygen challenge study, eight 9–11 week-old mice were randomized to either be kept normally or exposed to 95% oxygen for 72 hrs. All mice were then anesthetized, lavaged and sacrificed as above and lung tissue was prepared.

### Alveolar Epithelial Cells Isolation

Primary alveolar epithelial type II (ATII) cells were isolated from mice as previously described [10]. In brief, lungs were lavaged 2x with 1 ml sterile PBS, tissues digested with dispase and minced. The suspension was sequentially filtered through 100-, 20-, and 10-µm nylon meshes and centrifuged at 200 × g for 10 min. The pellet was resuspended in DMEM and a negative selection for lymphocytes/macrophages was performed by incubation on CD16/32- and CD45-coated Petri dishes for 30 min at 37°C. Negative selection for fibroblasts was performed by adherence for 45 min on cell culture dishes. Cell purity and viability was analysed in freshly isolated ATII cells directly after isolation. Cell purity was routinely assessed by epithelial cell morphology and immunofluorescence analysis of panCK, pro-SPC (both positive), αSMA, and CD45 (both negative) of cytocentrifuge preparations of ATII cells. Cell viability was checked by Trypan Blue exclusion. ATII cells used throughout this study demonstrated a 95±3% purity and >97% viability.

### RNA Extraction and RT-PCR from Mouse Lung Tissue and Cell Lines

RNA extraction with RNAzol followed the manufacturer’s protocol (WAK-Chemie Medical, Steinbach, Germany). RNA extracted from the above process was further purified using the RNAeasy protect minikit (Qiagen, Valencia, CA). cDNA was made using the First strand CDNA synthesis kit (GE healthcare, Piscataway, NJ). RNA was also extracted from A549, Calu-3 and HEK-29 cell lines and *SFTA2* q-PCR analysis performed with normalization for GAPDH. Primers used were: SFTA2 - forward: GGAGTCTTTTCTGACAAATTCCTC and reverse: GGTGTTGAGATCTTGCATGGTGG.

Real-time PCR from mouse lung tissue was carried out to determine the effect of different challenges on the levels of type-2 cell specific genes in the lung. The following genes were detected using the respective primers. SFTA2: Forward - CACACGCAGGGCCAAAGG Reverse - GAGGAGGCAGATCTTTTG; Sp-B: Forward - CACTGAGGATGCCATGGGCC Reverse - TGATCACAGACTTGCAGAAATGGCACT; Sp-C: Forward - CTTTCCTGTCCCGCTGCGGTT Reverse - GGCCTTGCTGTCAGCACCCTG; Sp-D: Forward - TCCTGGGCATCCTCAAAAGGCT Reverse - AGCGTCTAGAGGTTGCCTTCTCCC; Actin: Forward - GTGGGCCGCCCTAGGCACCA, Reverse – TGGCCTTAGGGTTCAGGG; HPRT – Forward - ATG TCC GGT AAC GGC GGC, Reverse - GGT ACA AGG CTT TCA GCA TCG C.

### Statistics

Statistical analyses employing the Mann–Whitney U test were performed with the software SPSS version 18.0 (Chicago, U.S.).

## Results

### Gene Characteristics of Human *SFTA2*

*SFTA2* is a single copy gene on chromosome 6 p21.33. It contains 3 exons with small introns such that the total genomic region corresponding to the transcript comprises only 826 bp. Interestingly, this region has been associated with diffuse panbronchiolitis (DPB) in Japanese patients [11]. *SFTA2* is positioned close to *DPCR1,* an unconfirmed candidate gene for DPB, *MUC21* (mucin 21), which encodes a mucin highly expressed in the lung, and *PBMUCL1* (panbronchiolitis related mucin-like 1), polymorphisms of which have been associated with DPB [12].

### Expression of SFTA2 in Human and Mouse Tissues

SFTA2 was identified to be highly expressed in the lung based on RNA-analysis using chip hybridization methods. In order to verify this data with an independent method, we investigated 48 human tissues by quantitative RT-PCR. While lung was the predominant organ, expression was also detected in cervix, esophagus, stomach, testis and kidney at levels above 1% of that found in the lung (Table 1). The following tissues or cells expressed less than 0.1% of the level found in the lung: duodenum, pituitary, trachea, ureter, adipose tissue, retina, prostate, vena cava, intracranial artery, placenta, epididymis, ovary, seminal vesicles, bone marrow, oviduct, tonsil, spinal cord, thymus, penis, colon, lymphocytes, urinary bladder, heart, optic nerve, small intestine, skeletal muscle, brain. SFTA2 is expressed in a number of human lung tumors but also tumors of the breast, ovary and cervix as well as tumor-derived cell lines (GeoProfiles).

**Table 1:** Relative *SFTA2* mRNA levels in human tissues normalized to GAPDH.

Tissue	Relative amount of mRNA normalized for GAPDH
Lung	100
Cervix	12.94
esophagus	12.85
stomach	3.42
Testis	2.02
Kidney	1.12
Rectum	1.02
pancreas	0.81
Vagina	0.50
lymph node	0.35
Uvula	0.34
pericardium	0,27
Thyroid	0.20
Skin	0.19
Liver	0.19
Tongue	0.19
uterine corpus	0.18
adrenal gland	0.15
Spleen	0.15
mammary gland	0.10

Out of 48 tissues investigated, those with mRNA levels above 0.1% of lung are listed. The relative amount of the lung was set to 100. The lung is the predominant site of expression, while there is expression >1% of lung also in cervix, esophagus, stomach, testis, kidney and rectum. Normalization for β-actin resulted in the same pattern.

In the mouse, SFTA2 was predominantly expressed in the lung while all other tissues investigated showed less than 1% of lung levels (data not shown).

### *SFTA2* Promoter Characteristics

A scan of promoter regions of *SFTA2* orthologues for known functional modules revealed two interesting combinations. A V$ETSF-V$SP1 combination ((V$ETSF: ETS1 factors; (EHF, ELF1through 3, ELF4, ELF5, ELK1, ELK3, ELK4, ERF, ERG, ETS1, ETS2, ETV1, ETV2, ETV3, ETV4, ETV5, ETV6, ETV7, FEV, FLI1, GABPA, GABPAP, GABPB1, GABPB2, SPDEF, SPI1, SPIB, SPIC), V$SP1F: GC-Box factors SP1/GC; (SP1 through 7; KLF10, KLF11 KLF16 KLF5)) was conserved in 7 species (hsa, mml, ptr, eca, cfa, bta, ssc), which has been reported to regulate the human Bruton’s agammaglobulinemia tyrosine kinase *(BTK*) [13], crucial in B-cell development. The second combination, V$ETSF-V$NFKB, (V$NFKB: Nuclear factor kappa B/c-rel (HIVEP1, HIVEP2, HIVEP3, NFKB1, NFKB2, REL, RELA, RELB) was conserved in 5 species (hsa, ptr, eca, bta, ssc), and has been identified as an important regulatory element for IRF8-dependent upregulation of *CCL5* as part of the innate immune response [14]. A highly conserved region of about 80 bp (identified by DiAlign2 [7]) 300 bp upstream of the human *SFTA2* transcription start site contains several potential binding sites for homeodomain factors, including *NKX-2-1* encoding thyroid transcription factor 1 (TTF-1), which plays an important role in lung development. These V$NKXH sites (V$NKXH: NKX homeodomain factors (HMX1, HMX2, HMX3, NKX2-1, NKX2-2, NKX2-3, NKX2-4, NKX2-5, NKX2-6, NKX2-8, NKX3-1, NKX3-2) are conserved in all 8 species and, except for sus scrofa, even in modules together with binding sites for Myc associated zinc fingers (V$MAZF MAZ, PATZ1), C2H2 zinc finger transcription factors 2 (V$ZF02: ZNF281, ZBTB7B, ZNF219, ZBTB7A, ZNF148, ZNF202, ZKSCAN3, ZNF300) or Krueppel-like transcription factors (V$KLFS: KLF1, KLF2, KLF3, KLF4, KLF6, KLF7, KLF8, KLF9, KLF12, KLF13, KLF15). All three binding site families have members with a role in the immune system (PATZ1, KLF6, ZNF148).

In the same region, however, 65 models containing 3 sites were identified with very specific distances due to the high evolutionary conversation. These models comprise 16 transcription factor binding site families. A scan through all human promoters using ModelInspector revealed a high specificity for transcription factor combinations with these specific distances. 35 are only present in the *SFTA2* promoter and less than 25 times in the whole human genome (between 4 and 22 times). The remaining 30 modules are found in promoters of additional genes (between 2 and 24 depending on the module). Among these are genes involved in lung development (SHH, GABRA1, GABRA3), lipid metabolism (LPL, CROT) and immune response (CD69, IL1RAP, BECN1) but no other surfactant proteins.

### SFTA2 Predicted Protein Characteristics

Human SFTA2 is a small peptide. The primary translation product comprises 78 amino acids. Cleavage of a 19 bp signal gives rise to a mature peptide of 59 amino acids (predicted molecular weight 6.55 kDa). SFTA2 is not hydrophobic such as surfactant proteins B or C.

**Figure 1:**
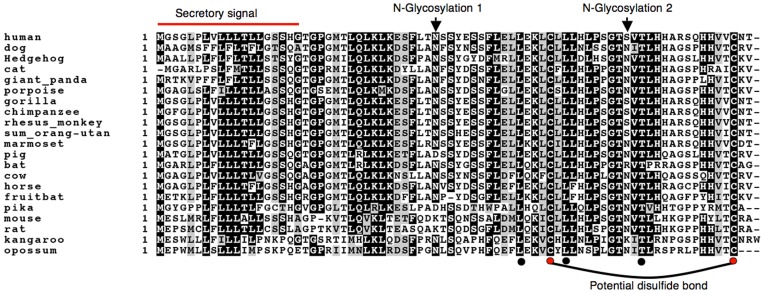
Alignment of SFTA2 amino acid sequences from 21 mammalian species. Secretory and glycosylation signals are recognized in all species. Shaded amino acid residues indicate a conservation of >70% (black: identical; grey: conserved). All species missing N-glycosylation signal 1 (position 37; pig, pika, mouse rat) have a signal at position 61. While only five amino acid residues are identical in all species (black dots), the only two cysteine residues are among them (red dots).

Orthologues were easily identified in all mammals including marsupials due to a high degree of conservation (Fig.1). Chromosomal localizations in syntenic regions confirmed the identity of orthologues for which chromosomal localizations were known. No other sequence-related proteins were identified in the databases. In addition to the signal peptide, two regions showed particularly high conservation (AA 25–33 and AA 44–66; Fig. 1). A helical structure could be predicted between AA 44 and 55. However, no similarity of these regions to known domains shared by other proteins was recognized.

Potential N-glycosylation signals were identified as depicted in Fig.1. The signal present in the human sequence at position 37 was missing in other animals such as pika, mouse or rat. However, all of these other species showed a glycosylation site at the position corresponding to the human AA 62 (Fig.1). In the amino acid sequence alignment of 21 species only 5 amino acid positions were fully conserved, and this was true for both the cysteine residues (Fig. 1). This and a prediction by the DiANNA software suggest a disulfide bond between C51 and C76.

### SFTA2 is Located in Intracellular Vesicles but not in Lamellar Bodies

**Figure 2:**
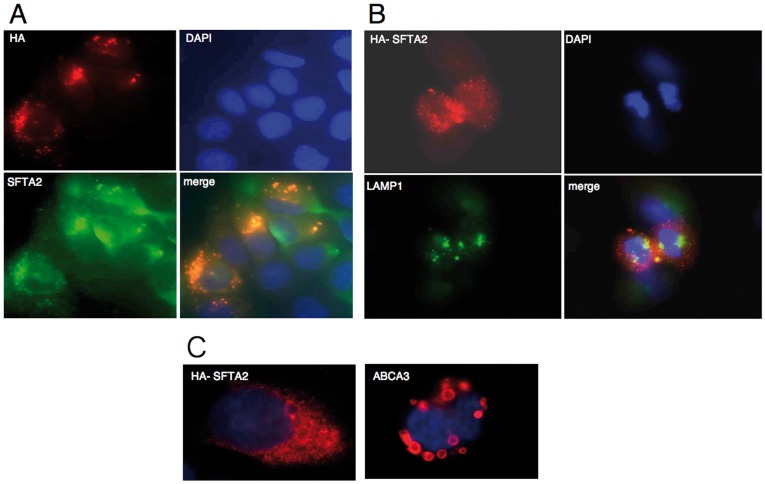
Immunofluorescence microscopic analyses in A549 (A, B) and MLE12 (C) cells after transfection with pCDNA-SFTA2-HA . **A.** Cells were analyzed with an HA epitope antibody and an SFTA2-specific peptide antibody. Colocalization of HA and SFTA2 signals indicates that our SFTA2 antibody recognizes human SFTA2 resulting from transfection. The punctate cytoplasmic pattern is consistent with localization in secretory vesicles. The background signal of non-transfected cells may have resulted from natural SFTA2 expression in A549 cells. **B.** SFTA2-HA signals do not colocalize with lysosomes or lysosome-related organelles such as lamellar bodies as indicated by LAMP1 immunostaining. **C.** In MLE12 cells, transfection with a plasmid construct encoding the phospholipid transporter ABCA3 labels the outer membrane of lamellar bodies as ring-like structures while epitope-tagged SFTA2 yields a distinctly different punctate cytoplasmic pattern.

In immunofluorescence microscopy of cultured SFTA2-HA transfected cells, we could clearly identify colocalization of the signal arising from the peptide antibody with the signal arising from the HA-epitope antibody, indicating that the peptide antibody recognized the protein arising from transfection (Fig. 2A). Subcellular localization was in small cytoplasmic compartments, consistent with secretory vesicles. LAMP1, a marker of lamellar bodies and lysosome-related organelles, did not show colocalization with SFTA2 in A549 cells (Fig. 2B). Furthermore, using ABCA3 as a lamellar body marker in MLE12 cells a pattern distinctly different from lamellar bodies was found for SFTA2 (Fig. 2C).

### SFTA2 Colocalizes with Golgi Apparatus and Secretory Vesicle Markers

**Figure 3:**
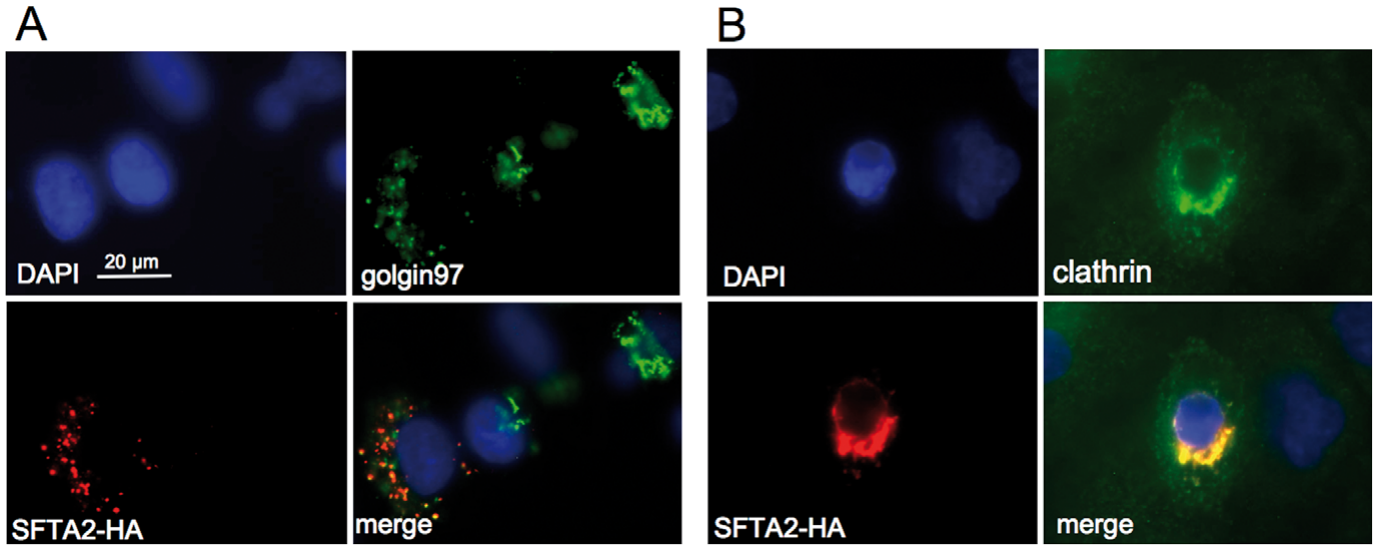
Immunofluorescence microscopic analysis of A549 cells transfected with an HA epitope-tagged SFTA2 expression plasmid. A. Partial colocalization with golgin97 indicates passage of SFTA2 through the Golgi apparatus. **B.** Partial colocalization with clathrin indicates presence of SFTA2 in secretory vesicles consistent with the classical pathway of secretion.

Colocalization experiments in transiently transfected A549 cells with golgin-97, a marker for Golgi apparatus, anti-clathrin antibody, a marker for secretory vesicles, and HA-epitope showed partial colocalisation of HA-antibody with golgin-97 and anti-clathrin antibody (Fig. 3) indicating that SFTA2 is a secretory protein and its subcellular localization is consistent with the classical secretory pathway.

### SFTA2 is an N-glycosylated Protein

**Figure 4:**
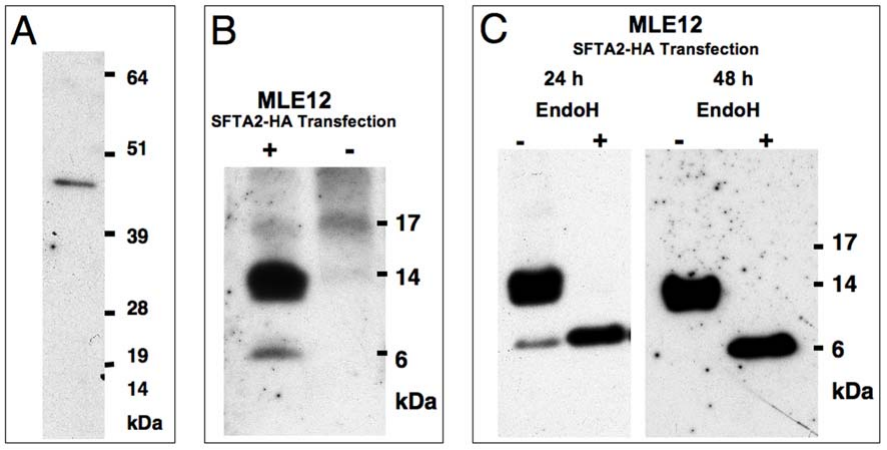
Immunoblot analysis and N-glycosylation testing of human SFTA2 . **A.** Anti-human SFTA2 peptide antibodies detect 50 ng of SFTA2-MBP fusion protein with an expected molecular weight of 48.5 kDa. **B.** 24 hrs after transfection with pCDNA3-SFTA2-HA, specific bands are detected at the expected 6.5 kDa, but also at 13 kDa (+ transfected, - non-transfected MLE12 cells). **C.** MLE12 cells were harvested 24 h (left) or 48 h (right) after transfection. While after 24 hrs the 6 kDa band is still present, it has disappeared after 48 h. Deglycosylation using endo-β-N-acetylglucosaminidase shifts the 13 kDa band to 6 kDa demonstrating that the 13 kDa band represents a N-glycosylated form.

The predicted molecular weight of SFTA2 is 6.55 kDa. While our peptide antibody recognized the transfected (Fig. 2A) and bacterially expressed recombinant protein (Fig. 4A), the affinity for the antigen in immunoblot analyses was apparently not sufficient to detect the natural protein in lung and A549 homogenates. Transfection of MLE12 cells with the SFTA2-HA construct resulted in specific bands at 13 kDa and 6 kDa, if cells were harvested after 24 h. After 48 h only the 13 kDa band was detected (Fig. 4 B, C). This would be consistent with a shift of the apparent molecular weight (retardation in polyacrylamide gel electrophoresis) due to glycosylation of the protein. With Endo H treatment, the 13 kDa band disappeared and only the predicted band at 6 kDa was detected, which corresponds to the deglycosylated peptide.

### SFTA2 is Highly Expressed in Type II Pneumocytes and Nonciliated Bronchiolar Epithelial Cells

**Figure 5:**
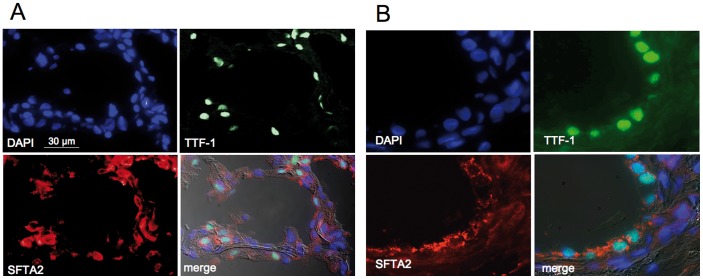
Frozen section immunofluorescence microscopic analysis of SFTA2 in the human lung. While DAPI stains all nuclei, TTF-1 (thyroid transcription factor-1) antibodies label nuclei of type II pneumocytes and nonciliated bronchiolar epithelial cells only. **A.** The SFTA2 polyclonal peptide antibody demonstrates cytoplasmic staining localizing to the same cells as TTF-1 in alveoli (indicating SFTA2 expression restricted to type II pneumocytes in the alveolus). **B.** SFTA2 signals also colocalize with TTF-1 in nonciliated bronchiolar epithelium. Merge: superimposition of all exposures including differential interference contrast to visualize tissue morphology.

In frozen lung sections, immunfluorescence staining of SFTA2 by our peptide antibody detected specific signals in the cytoplasm of alveolar epithelial cells and bronchiolar cells. When peptide antibodies were blocked by preabsorption with the peptides used for immunization in 10-fold molar excess, immunofluorescence signals were nearly abolished (Fig. S1). In order to define the cell types, we double-stained human lung tissues with SFTA2 and TTF-1 specific antibodies. In the alveolar eptihelium, TTF-1 specifically labels nuclei of type II pneumocytes. and nonciliated epithelial cells of the terminal bronchiolus [15]. As shown in Fig. 5, cytoplasmic signals corresponding to SFTA2 were exclusively detected in cells expressing TTF-1 in their nuclei, thus identified as type II pneumocytes in the alveolus (Fig. 5A) and nonciliated epithelium in bronchioli (Fig. 5B).

### RT-PCR Studies in Isolated Type-II Cells and Cell Lines

**Figure 6:**
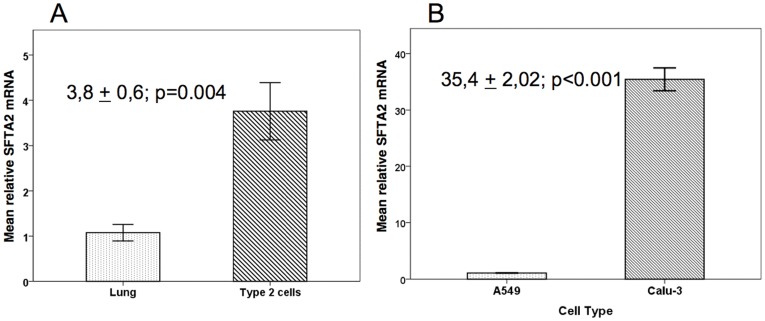
Quantitative RT-PCR-analysis of SFTA2 mRNA. Relative abundance was set to 1 for lung tissue and A549 cells respectively. **A.** Isolated mouse type II cells demonstrated a 3.8 fold enrichment of SFTA2 messenger RNA compared to total lung tissue. **B.** SFTA2 mRNA is 35.4 times more abundant in Calu-3 cells than in A549 cells. While A549 human adenocarcinoma cells show some characteristics of Type II cells, Calu-3 is an airway adenocarcinoma cell line that expresses submucosal gland cell features.

*SFTA2* expression was found to be 4-fold in isolated type II cells of the mouse lung as compared to whole lung homogenate. In addition, among the cell lines investigated, SFTA2 expression was highest in Calu-3 cells (35.4 fold) as compared to A549 (Fig.6) and not detected in HEK-29 cells (not shown).

### *SFTA2* is Downregulated by LPS-induced Inflammation, but not by Hyperoxia

**Figure 7:**
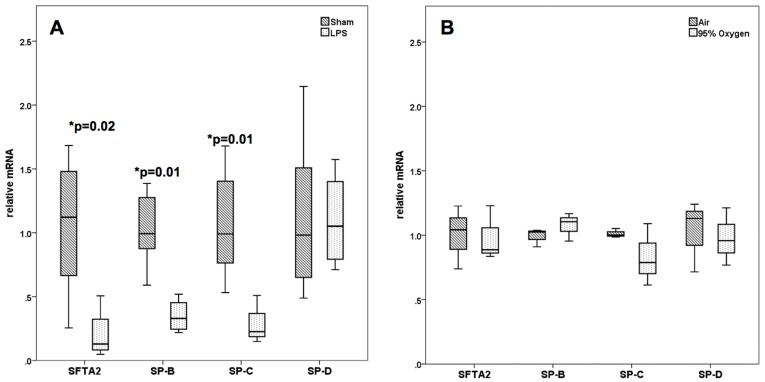
Analyses of specific mRNA levels in mouse lung in response to challenges by lipopolysaccharide instillation or oxygen exposure. The mean values of untreated animals were set to 1.0. **A.** Sfta2 qRT-PCR of mouse lung tissue 18 h after tracheal LPS instillation in relation to other type II pneumocyte proteins SP-B, SP-C, SP-D. Significant SFTA2 downregulation was observed in LPS-treated mice paralleling SP-B and SP-C, but not SP-D. **B.** Analysis of the same transcripts after room air vs. 95% oxygen exposure. We observed alteration of SFTA2 and any of the other messages despite significant inflammatory reaction (not shown).

Since SFTA2 is expressed by the alveolar and nonciliated bronchiolar epithelium and most likely secreted into the alveolar space, we had speculated about a role as a messenger involved in inflammation and defence. In an intratracheal LPS challenge model of C57BL/6 mice, we observed a clear downregulation of *Sfta2* paralleling other proteins secreted by the type II cells such as SP-B or SP-C, but not SP-D, indicating that this downregulation was not solely due to damage to type II pneumocytes (Fig. 7A). Hyperoxia for 72 hours showed no such change (Fig. 7B), although the inflammatory reaction of the lung due to hyperoxia was clearly demonstrated by lavage analysis (not shown).

## Discussion

We have identified *SFTA2* as a novel transcript highly expressed in the lung. In all mammals with available genomes, the encoded peptide is unique in the sense that there is no homologue within a species nor are there any recognizable domain structures shared by other proteins. SFTA2 is a glycosylated hydrophilic surfactant protein with a cleavable secretory signal at the amino terminus highly expressed in type II pneumocytes and nonciliated bronchiolar epithelium. N-glycosylation may be required for stabilization of the peptide and a disulfide bond appears likely to be present.

Secretion of SFTA2 into extracellular space was not demonstrated here. However, the presence of a specific cleavable secretory signal in SFTA2 sequence as well as its targeting to cytoplasmic vesicles partially colocalizing with golgin97 and clathrin indicate the passage SFTA2 through the classical pathway of secretion. Specific triggers are probably needed for secretion.

The *in silico* promoter analysis identified several conserved binding sites within the *SFTA2* promoter. Although these are frequent sites, combinations with the identified distances are rather rare. Together with the unique amino acid sequence, we regard this as an indication of unique regulation and function of SFTA2. It is striking that SFTA2 expression in the lung parallels the expression of TTF-1. This is consistent with TTF-1 binding sites identified in the SFTA2 promoter and may indicate that TTF-1 might regulate SFTA2 expression especially in the lung.

Proteins secreted by type II pneumocytes and nonciliated bronchiolar epithelium may serve various functions. While the small hydrophobic proteins SP-B and SP-C are vital for the physical properties of pulmonary surfactant and thereby lung expansion [16], [17], the hydrophilic proteins SP-A and SP-D confer innate immunity promoting phagocytosis by macrophages [18]. Furthermore, SP-A is involved in the control of surfactant production and both SP-A and SP-D have a role in limiting excessive inflammation [19], [20], [21]. We have demonstrated that SFTA2 is neither localized within lysosomal-related organelles nor is its expression restricted to type II cells. Therefore, it appears unlikely that SFTA2 (like SP-B or SP-C) is directly involved in the determination of physical properties of surfactant. Moreover, we show that SFTA2 is differentially expressed in response to lung inflammation induced by smooth LPS, whereas no change is observed in a hyperoxia model. While alveolar macrophages play a role in the reaction to LPS, triggering inflammation, in hyperoxia direct damage to endothelial and type I cells results in inflammation. Interestingly, SFTA2 - like SP-A [22] and SP-D [23] - is also expressed in the female genital tract. The identification of a specific receptor for SFTA2 in certain lung cell types (and the female genital tract) will add significantly to our understanding.

SFTA2 defects may be directly involved in human lung disease. The *SFTA2* locus has been implicated in diffuse panbronchiolitis (DPB). Possible mechanisms are as yet unclear. In the past, knockout mouse models have significantly contributed to the understanding of the function of surfactant proteins [24], [25], [26]. Therefore, further studies may involve targeting of Sfta2 in mice.

In conclusion, we have identified SFTA2 as a novel secretory protein predominantly expressed by lung type II pneumocytes and nonciliated bronchiolar cells. While SFTA2 function has not been directly addressed, preliminary data encourage speculations on a regulatory role in inflammation and defence.

## Supporting Information

Figure S1 **Lung frozen section immunofluorescence analysis.** Blocking of peptide antibody with a 10-fold molar excess of peptides. While in absence of blocking, the SFTA2 antibody led to distinct cytoplasmic staining (please see Fig. 4), blocking with the peptides used for antibody generation completely blocked the specific signal. **A.** Merge of all images. **B.** DAPI staining of all nuclei. **C.** TTF-1 staining labelling nuclei of type II cells and nonciliated bronchiolar epithelium. **D.** Signals from SFTA2 antibody in the presence of blocking peptides are nearly absent despite high exposure time. **E.** Differential interference contrast to demonstrate lung morphology.
(TIFF)
