# Supplementary figures and images for: SFTA2—A Novel Secretory Peptide Highly Expressed in the Lung—Is Modulated by Lipopolysaccharide but Not Hyperoxia
Source: PLoS One. 2012 Jun 29;7(6):e40011. doi: 10.1371/journal.pone.0040011 (PMC3386909; doi:10.1371/journal.pone.0040011)

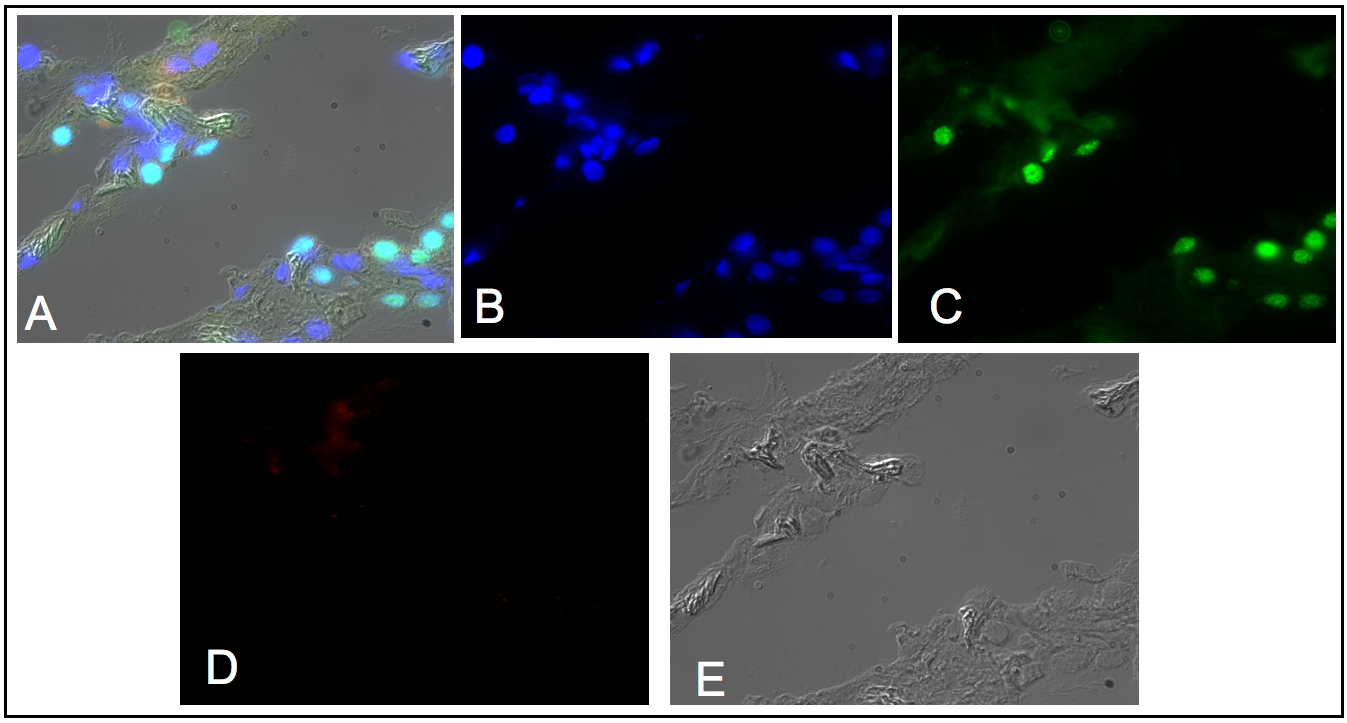

Supplement: Figure S1 — Lung frozen section immunofluorescence analysis. Blocking of peptide antibody with a 10-fold molar excess of peptides. While in absence of blocking, the SFTA2 antibody led to distinct cytoplasmic staining (please see Fig. 4), blocking with the peptides used for antibody generation completely blocked the specific signal. A. Merge of all images. B. DAPI staining of all nuclei. C. TTF-1 staining labelling nuclei of type II cells and nonciliated bronchiolar epithelium. D. Signals from SFTA2 antibody in the presence of blocking peptides are nearly absent despite high exposure time. E. Differential interference contrast to demonstrate lung morphology. (TIFF) [file pone.0040011.s001.tiff]
